# Three closely related (2*E*,2′*E*)-3,3′-(1,4-phenyl­ene)bis­[1-(meth­oxy­phen­yl)prop-2-en-1-ones]: supra­molecular assemblies in one dimension mediated by hydrogen bonding and C—H⋯π inter­actions

**DOI:** 10.1107/S2056989017007460

**Published:** 2017-05-26

**Authors:** Aijia Sim, C. S. Chidan Kumar, Huey Chong Kwong, Li Yee Then, Yip-Foo Win, Ching Kheng Quah, S. Naveen, S. Chandraju, N. K. Lokanath, Ismail Warad

**Affiliations:** a X-ray Crystallography Unit, School of Physics, Universiti Sains Malaysia, 11800 USM, Penang, Malaysia; b Department of Engineering Chemistry, Vidya Vikas Institute of Engineering &, Technology, Visvesvaraya Technological University, Alanahalli, Mysuru 570 028, Karnataka, India; c School of Chemical Sciences, Universiti Sains Malaysia, Penang 11800 USM, Malaysia; d Department of Chemical Science, Faculty of Science, Universiti Tunku Abdul, Rahman, Perak Campus, Jalan Universiti, Bandar Barat, Perak, Malaysia; e Institution of Excellence, University of Mysore, Manasagangotri, Mysuru 570 006, India; f Department of Sugar Technology & Chemistry, University of Mysore, Sir M. Visvesvaraya PG Centre, Tubinakere, Mandya 571 402, India; g Department of Studies in Physics, University of Mysore, Manasagangotri, Mysuru, 570 006, India; h Department of Chemistry, Science College, An-Najah National University, PO Box 7, Nablus, West Bank, Palestinian Territories

**Keywords:** crystal structure, bis­chalcone, meth­oxy­phenyl ring, enone bridge, C—H⋯O hydrogen bond, C—H⋯π inter­actions

## Abstract

In the title compounds, (2*E*,2′*E*)-3,3′-(1,4-phenyl­ene)bis­[1-(2-meth­oxy­phen­yl)prop-2-en-1-one], C_26_H_22_O_4_ (I), (2*E*,2′*E*)-3,3′-(1,4-phenyl­ene)bis­[1-(3-meth­oxy­phen­yl)prop-2-en-1-one], C_26_H_22_O_4_ (II) and (2*E*,2′*E*)-3,3′-(1,4-phenyl­ene)bis­[1-(3,4-di­meth­oxy­phen­yl)prop-2-en-1-one], C_28_H_26_O_6_ (III), the asymmetric unit consists of a half-mol­ecule, completed by crystallographic inversion symmetry. The dihedral angles between the central and terminal benzene rings are 56.98 (8), 7.74 (7) and 7.73 (7)° for (I), (II) and (III), respectively. In the crystal of (I), mol­ecules are linked by pairs of C—H⋯π inter­actions into chains running parallel to [101]. The packing for (II) and (III), features inversion dimers linked by pairs of C—H⋯O hydrogen bonds, forming *R*_2_^2^(16) and *R*_2_^2^(14) ring motifs, respectively, as parts of [201] and [101] chains, respectively.

## Abstract 2

The three title compounds are centrosymmetric. Their packing features chains linked by either pairwise C—H⋯O or C—H⋯π inter­actions.

## Chemical context   

Chalcones and their derivatives are natural or synthetic 1,3-diaryl-2-propenones that may exist in *cis* and *trans* isomeric forms, the *trans* form being thermodynamically stable. The *α*,*β*-unsaturated enone C=C—C(=O)—C moiety in the chalcone structure plays an important role in the biological activities of these species (Husain *et al.*, 2013 ▸; Abdel Ghani *et al.*, 2008 ▸). As a result of the -enone system, these mol­ecules present relatively low redox potentials and have a greater probability of undergoing electron-transfer reactions. Apart from the biological activities, the photophysical properties of chalcone derivatives also attracted considerable attention from both chemists and physicists. Many chalcone derivatives have been reported in relation to non-linear optics (NLO), photorefractive polymers, holographic recording materials and fluorescent probes for sensing metal ions (Ruzié *et al.*, 2009 ▸; Wei *et al.*, 2011 ▸; Chandra Shekhara Shetty *et al.*, 2016 ▸). As part of our studies in this area, we report herein the syntheses and structures of three new bis­chalcone derivatives, (2*E*,2′*E*)-3,3′-(1,4-phenyl­ene)bis­(1-(2-meth­oxy­phen­yl)prop-2-en-1-one), C_26_H_22_O_4_ (I), (2*E*,2′*E*)-3,3′-(1,4-phenyl­ene)bis­(1-(3-meth­oxy­phen­yl)prop-2-en-1-one), C_26_H_22_O_4_ (II) and (2*E*,2′*E*)-3,3′-(1,4-phenyl­ene)bis­(1-(3,4-di­meth­oxy­phen­yl)prop-2-en-1-one), C_28_H_26_O_6_ (III).
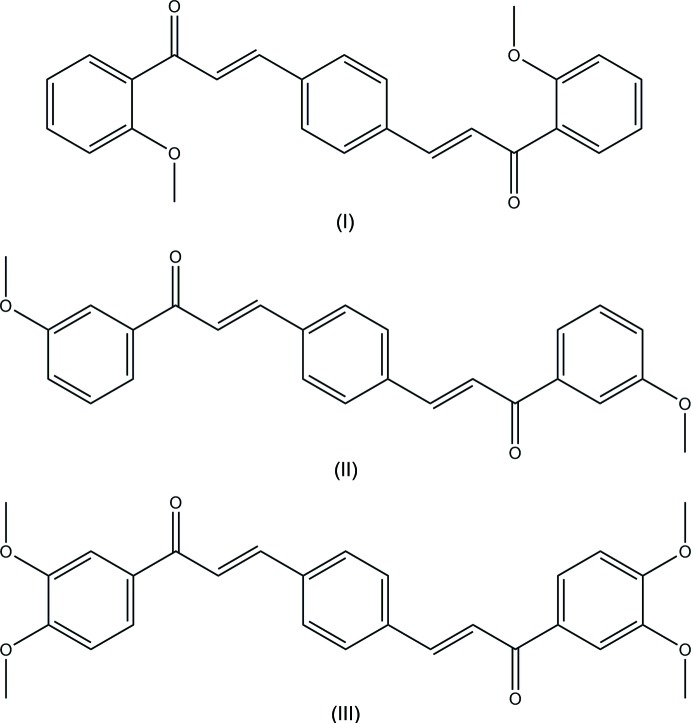


## Structural commentary   

**Figure 1:**
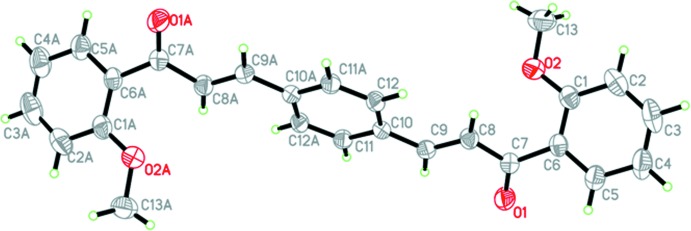
The mol­ecular structure of (I), showing 40% probability displacement ellipsoids. [Symmetry code: (*A*) −*x*, 1 − *y*, −*z*.]

The mol­ecular structures of (I)–(III) are shown in Figs. 1 ▸–3 ▸ ▸, respectively. The asymmetric unit of each compound consists of a half-mol­ecule, with the complete mol­ecule generated by a crystallographic inversion centre at the centroid of the central benzene ring.

Each compound is constructed from two aromatic rings (centre benzene and terminal meth­oxy­phenyl rings), which are linked by a C=C—C(=O)—C enone bridge. Despite having an extra meth­oxy substituent, the conformation of compounds (II) and (III) are very similar, as indicated by the dihedral angles between the rings of 7.74 (7) and 7.73 (7)°, respectively. The enone linkage moiety of compounds (II) and (III) has similar torsion angles [O1—C7—C8—C9 = 0.2 (2) and 7.3 (2)°, respectively], but compound (II) has a higher overall planarity than compound (III), as its enone bridge forms a smaller torsion angle with the meth­oxy­phenyl ring [C1—C6—C7—O1 = −6.5 (2)°] and benzene ring [C8—C9—C10—C11 = −1.7 (2)°; C1—C6—C7—O1 = 7.3 (2)° and C8—C9—C10—C11 = −5.6 (2)° in (III)]. Compared to the nearly coplanar arrangement of rings in compounds (II) and (III), compound (I) is substanti­ally twisted [O1—C7—C8—C9 = −13.5 (2)° and C1—C6—C7—O1 = 143.60 (15)°] about the enone bridge, which may arise from steric repulsion with the *ortho*-O2 atom. Hence, the dihedral angle between the 2-meth­oxy­phenyl and benzene rings in (I) increases to 56.98 (8)°. Key torsion angles are tabulated in Table 1 ▸. The C atoms of the meth­oxy groups are close to the planes of their attached rings in all cases: for (I), deviation of C13 = 0.163 (2) Å, for (II), deviation of C13 = 0.329 (2) Å, and for (III), deviations of C13 and C14 = 0.091 (2) and −0.266 (2) Å, respectively.

## Supra­molecular features   

**Figure 4:**
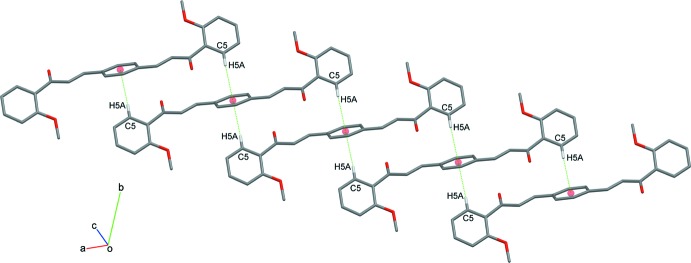
Fragment of a [101] chain of mol­ecules of (I) linked by pairs of weak C—H⋯π inter­actions (dashed lines).

**Figure 5:**
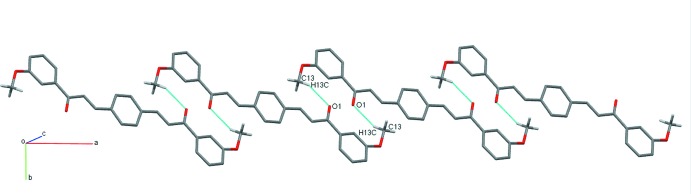
Fragment of a [201] chain of mol­ecules of (II) linked by pairs of weak C—H⋯O inter­actions (dashed lines).

**Figure 6:**
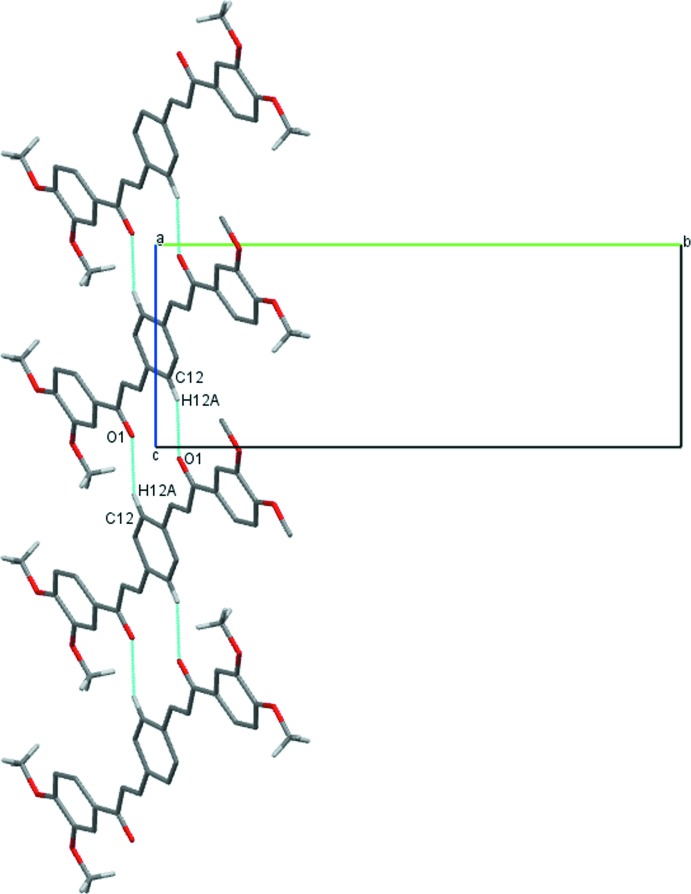
Fragment of a [101] chain of mol­ecules of (III) linked by pairs of weak C—H⋯O inter­actions (dashed lines).

The packing of (I) is consolidated by a weak C—H⋯π contact (Table 2 ▸) involving a hydrogen atom from the phenyl ring and the centroid of the central benzene ring (C10–C12/C10*A*–C12*A*). This C—H⋯π inter­action connects the mol­ecules of (I) into chains parallel to the [101] direction with a C—H⋯π distance of 2.74 Å (Fig. 4 ▸). In the supra­molecular assemblies of compounds (II) and (III), the mol­ecules are connected by pairs of C—H⋯O hydrogen bonds (Table 2 ▸) into inversion dimers, which form 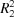
(16) and 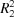
(14) ring motifs, respectively. The dimers are parts of [201] chains (Fig. 5 ▸) in (II), while mol­ecules in compound (III) are parts of chains propagating in the [101] direction (Fig. 6 ▸).

## Database survey   

A search of the Cambridge Structural Database (CSD, Version 5.38, last update November 2016; Groom *et al.*, 2016 ▸) using (2*E*,2′*E*)-3,3′-(1,4-phenyl­ene)bis­(1-phenyl­prop-2-en-1-one) as the main skeleton, revealed the presence of four structures containing the bis­chalcone moiety with different substituents, similar to the title compounds in this study. These include 3,3′-(1,4-phenyl­ene)bis­[1-(*X*)prop-2-en-1-one], where *X* = 2-hy­droxy­phenyl (DIDNUB; Gaur & Mishra, 2013 ▸), 4-chloro­phenyl (KIKFUG; Harrison *et al.*, 2007 ▸), 4-meth­oxy­phenyl (UDUPUF[Chem scheme2]; Harrison *et al.*, 2007*a* ▸) and 3,4-meth­oxy­phenyl (UDUQAM[Chem scheme2]; Harrison *et al.*, 2007*b* ▸). It is notable that UDUPUF[Chem scheme2] and UDUQAH[Chem scheme2] are positional isomers of compounds (I) and (II), and (III), respectively, differing from them only in the location of the meth­oxy substituent (see scheme below[Chem scheme2]). The dihedral angles between the central and terminal phenyl ring in these compounds vary from 10.9 to 46.3°. In terms of the title compounds, (II) and (III) are more planar [7.74 (7) and 7.73 (7)°] while compound (I) is more twisted [56.98 (8)°]. The supra­molecular assembly in UDUPUF[Chem scheme2] also depends upon a single C—H⋯O hydrogen bond between inversion-related pairs of mol­ecules, forming a centrosymmetric dimer.
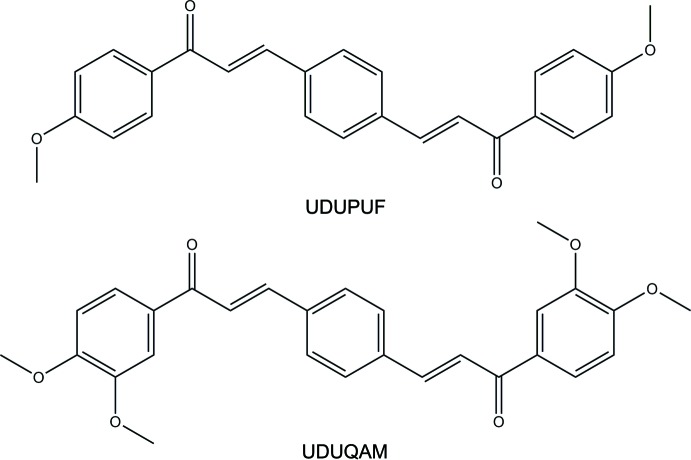


## Synthesis and crystallization   

A mixture of the corresponding meth­oxy­aceto­phenone (0.02 mol) and terephthaldi­aldehyde (0.01 mol) was dissolved in methanol (20 ml). A catalytic amount of NaOH was added to the solution dropwise with vigorous stirring. The reaction mixtures were stirred for about 5–6 h at room temperature. The resultant crude products were filtered, washed successively with distilled water and recrystallized from ethanol to obtain the title compounds. Yellow blocks [(I) and (III)] and yellow needles (II) were recrystallized using the solvents noted below.

**(2** ***E*** **,2′** ***E*** **)-3,3′-(1,4-phenyl­ene)bis­(1-(2-meth­oxy­phen­yl)prop-2-en-1-one), C_26_H_22_O_4_ (I)**

Solvent for growing crystals: acetone; yield 85%, m.p. 429–431 K. FT–IR [ATR (solid) cm^−1^]: 3010 (Ar, C—H, *ν*), 2945 (methyl, C—H, *νs*), 2842 (methyl, C—H, *ν*), 1658 (C=O, *ν*), 1598, 1417(Ar, C=C, *ν*), 1245, 1055 (C—O, *ν*). ^1^H NMR (500 MHz, CDCl_3_): *δ* ppm 7.677–7.632 (*m*, 8H, ^5^CH, ^8^CH, ^11^CH, ^12^CH), 7.536–7.504 (*t*, 2H, *J* = 8.0 Hz ^3^CH), 7.496–7.437 (*d*, 2H, *J* = 15.9 Hz, ^9^CH), 7.095–7.065 (*t*, 2H, *J* = 8.0 Hz, ^4^CH), 7.048–7.031 (*d*, 2H, *J* = 8.0 Hz, ^2^CH), 3.944 (*s*, 6H, ^13^CH_3_). ^13^C NMR (125 MHz, CDCl_3_): *δ* ppm 192.61 (C7), 158.23 (C1), 141.48 (C9), 136.95 (C10), 133.09 (C3), 130.47 (C5), 129.17 (C6), 128.84 (C11, C12), 127.91 (C4), 120.84 (C8), 111.69 (C2), 55.80 (C13).

**(2** ***E*** **,2′** ***E*** **)-3,3′-(1,4-phenyl­ene)bis­(1-(3-meth­oxy­phen­yl)prop-2-en-1-one), C_26_H_22_O_4_ (II)**

Solvent for growing crystals: chloro­form and methanol; yield 85%, m.p. 444–446 K. FT–IR [ATR (solid) cm^−1^]: 3074 (Ar, C—H, *ν*), 2952 (methyl, C—H, *νs*), 2839 (methyl, C—H, *ν*), 1658 (C=O, *ν*), 1582, 1414 (Ar, C=C, *ν*), 1259, 1022 (C—O, *ν*). ^1^H NMR (500 MHz, CDCl_3_): *δ* ppm 7.855–7.823 (*d*, 2H, *J* = 15.7 Hz, ^8^CH), 7.722 (*s*, 4H, ^11^CH, ^12^CH) , 7.650–7.635 (*d*, 2H, *J* = 8.0 Hz, ^5^CH), 7.606–7.574 (*m*, 2H, ^1^CH, ^9^CH), 7.473–441 (*t*, 2H, *J* = 8.0 Hz, ^4^CH), 7.189–7.172 (*d*, 2H, *J* = 8.0 Hz, 3CH), 3.924 (*s*, 6H, ^13^CH_3_). ^13^C NMR (125 MHz, CDCl_3_): *δ* ppm 189.99 (C7), 159.99 (C2), 143.57 (C9), 139.46 (C10), 136.92 (C6), 129.65 (C5), 128.98 (C11, C12), 123.14 (C8), 121.08 (C3), 119.45 (C4), 112.96 (C1), 55.53 (C13)

**(2** ***E*** **,2′** ***E*** **)-3,3′-(1,4-phenyl­ene)bis­(1-(3,4-di­meth­oxy­phen­yl)prop-2-en-1-one), C_28_H_26_O_6_ (III)**

Solvent for growing crystals: acetone; yield 85%, m.p. 479–481 K. FT–IR [ATR (solid) cm^−1^]: 3018 (Ar, C—H, *ν*), 2962 (methyl, C—H, *νs*), 2836 (methyl, C—H, *ν*), 1651 (C=O, *ν*), 1592, 1418 (Ar, C=C, *ν*), 1240, 1017 (C—O, *ν*). ^1^H NMR (500 MHz, CDCl_3_): *δ* ppm 7.857–7.826 (*d*, 2H, *J* = 15.6 Hz, ^8^CH), 7.740–7.720 (*m*, 6H, ^5^CH, ^11^CH, ^12^CH), 7.666 (*s*, 2H, ^1^CH), 7.651–7.619 (*d*, 2H, *J* = 15.6 Hz, ^9^CH), 6.984–6.967 (*d*, 2H, *J* = 8.4 Hz, ^4^CH), 4.012–4.009 (*d*, 12H, ^13^CH_3_, ^14^CH_3_). ^13^C NMR (125 MHz, CDCl_3_): *δ* ppm 188.34 (C7), 153.47 (C3), 149.37 (C2), 142.80 (C9), 136.94 (C10), 131.22 (C6), 128.89 (C11, C12), 123.11 (C5), 122.62 (C8), 110.82 (C4), 110.01 (C1), 56.15 (C14), 66.10 (C13)

## Refinement   

Crystal data, data collection and structure refinement details are summarized in Table 3 ▸. In (I), (II) and (III), the C-bound H atoms were positioned geometrically [C—H = 0.93–0.96 Å] and refined using a riding model with *U*_iso_(H) = 1.5*U*_eq_(C-meth­yl) and 1.2*U*_eq_(C) for other H atoms.

## Supplementary Material

Crystal structure: contains datablock(s) global, I, II, III. DOI: 10.1107/S2056989017007460/hb7678sup1.cif

Structure factors: contains datablock(s) I. DOI: 10.1107/S2056989017007460/hb7678Isup2.hkl

Structure factors: contains datablock(s) II. DOI: 10.1107/S2056989017007460/hb7678IIsup3.hkl

Structure factors: contains datablock(s) III. DOI: 10.1107/S2056989017007460/hb7678IIIsup4.hkl

Supporting information file. DOI: 10.1107/S2056989017007460/hb7678Isup5.cml

Supporting information file. DOI: 10.1107/S2056989017007460/hb7678IIsup6.cml

Supporting information file. DOI: 10.1107/S2056989017007460/hb7678IIIsup7.cml

CCDC references: 1449626, 1449625, 1449624

Additional supporting information:  crystallographic information; 3D view; checkCIF report

## Figures and Tables

**Figure 2:**
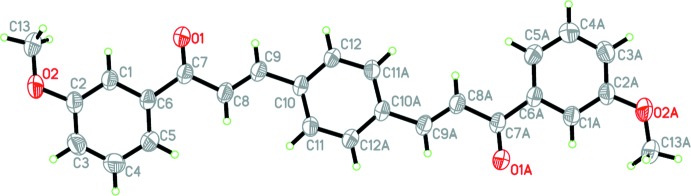
The mol­ecular structure of (II), showing 40% probability displacement ellipsoids. [Symmetry code: (*A*) 1 − *x*, 1 − *y*, 1 − *z*.]

**Figure 3:**
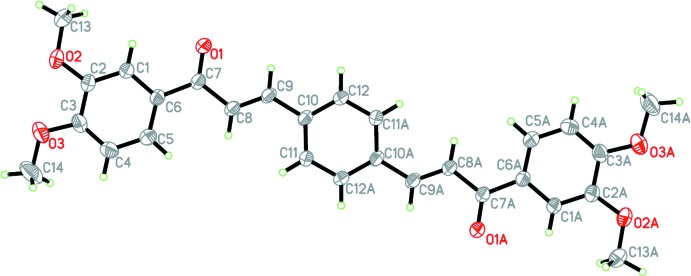
The mol­ecular structure of (III), showing 40% probability displacement ellipsoids. [Symmetry code: (*A*) 2 − *x*, −*y*, 1 − *z*.]

**Table 1:** Selected torsion and dihedral angles (°) for compounds (I)–(III) Dihedral 1 is the dihedral angle between the mean planes of the terminal meth­oxy­phenyl and central benzene rings.

Compound	C1—C6—C7—O1	O1—C7—C8—C9	C8—C9—C10—C11	Dihedral 1
(I)	−13.5 (2)	143.60 (15)	167.44 (15)	56.98 (8)
(II)	0.2 (2)	6.5 (2)	−1.6 (2)	7.74 (7)
(III)	7.3 (2)	7.3 (2)	−5.6 (2)	7.73 (7)

**Table 2:** Hydrogen-bonding geometry (Å, °) for compounds (I)–(III) *Cg*1 is the centroid of the C10–C12/C10*A*–C12*A* ring.

Compound	D—H⋯A	D—H	H⋯A	D⋯A	D—H⋯A
(I)	C5—H5*A*⋯*Cg*1^i^	0.93	2.74	3.491 (3)	139
(II)	C13—H13*C*⋯O1^ii^	0.96	2.60	3.503 (3)	157
(III)	C12—H12*A*⋯O1^iii^	0.96	2.47	3.337 (3)	156

Symmetry codes: (i) 1 + *x*, *y*, 1 + *z*; (ii) 3 − *x*, 1 − *y*, 2 − *z*; (iii) 1 − *x*, −*y*, −*z*.

**Table 3:** Experimental details

	(I)	(II)	(III)
Crystal data			
Chemical formula	C_26_H_22_O_4_	C_26_H_22_O_4_	C_28_H_26_O_6_
*M*_r_	398.43	398.43	458.49
Crystal system, space group	Monoclinic, *P*2_1_/*c*	Monoclinic, *P*2_1_/*c*	Monoclinic, *P*2_1_/*n*
Temperature (K)	294	294	294
*a*, *b*, *c* (Å)	7.1078 (11), 24.544 (4), 6.0449 (9)	5.2626 (5), 15.6157 (14), 12.4824 (11)	6.9595 (6), 21.0272 (17), 8.3297 (7)
β (°)	101.898 (2)	98.760 (2)	103.602 (2)
*V* (Å^3^)	1031.9 (3)	1013.83 (16)	1184.77 (17)
*Z*	2	2	2
Radiation type	Mo *K*α	Mo *K*α	Mo *K*α
μ (mm^−1^)	0.09	0.09	0.09
Crystal size (mm)	0.39 × 0.35 × 0.18	0.90 × 0.48 × 0.09	0.35 × 0.27 × 0.16

Data collection			
Diffractometer	Bruker APEXII DUO CCD area-detector	Bruker APEXII DUO CCD area-detector	Bruker APEXII DUO CCD area-detector
Absorption correction	Multi-scan (*SADABS*; Bruker, 2012 ▸)	Multi-scan (*SADABS*; Bruker, 2012 ▸)	Multi-scan (*SADABS*; Bruker, 2012 ▸)
*T*_min_, *T*_max_	0.883, 0.985	0.874, 0.992	0.890, 0.985
No. of measured, independent and observed [*I* > 2σ(*I*)] reflections	8322, 2183, 1705	17791, 2458, 1574	12532, 3165, 2328
*R*_int_	0.024	0.043	0.030
(sin θ/λ)_max_ (Å^−1^)	0.634	0.662	0.683

Refinement			
*R*[*F* ^2^ > 2σ(*F* ^2^)], *wR*(*F* ^2^), *S*	0.040, 0.119, 1.03	0.044, 0.132, 1.03	0.049, 0.136, 1.04
No. of reflections	2183	2458	3165
No. of parameters	137	137	156
H-atom treatment	H-atom parameters constrained	H-atom parameters constrained	H-atom parameters constrained
Δρ_max_, Δρ_min_ (e Å^−3^)	0.19, −0.14	0.14, −0.14	0.20, −0.16

Computer programs: *APEX2* and *SAINT* (Bruker, 2012 ▸), *SHELXS97* (Sheldrick, 2008 ▸), *SHELXL2013* (Sheldrick, 2015 ▸), *Mercury* (Macrae *et al.*, 2006 ▸) and *PLATON* (Spek, 2009 ▸).

## supplementary crystallographic information

### (I) (2E,2'E)-3,3'-(1,4-Phenylene)bis[1-(2-methoxyphenyl)prop-2-en-1-one] Crystal data

**Table:**

C_26_H_22_O_4_	*F*(000) = 420
*M**_r_* = 398.43	*D*_x_ = 1.282 Mg m^−^^3^
Monoclinic, *P*2_1_/*c*	Mo *K*α radiation, λ = 0.71073 Å
*a* = 7.1078 (11) Å	Cell parameters from 3004 reflections
*b* = 24.544 (4) Å	θ = 2.9–26.4°
*c* = 6.0449 (9) Å	µ = 0.09 mm^−^^1^
β = 101.898 (2)°	*T* = 294 K
*V* = 1031.9 (3) Å^3^	Block, yellow
*Z* = 2	0.39 × 0.35 × 0.18 mm

### (I) (2E,2'E)-3,3'-(1,4-Phenylene)bis[1-(2-methoxyphenyl)prop-2-en-1-one] Data collection

**Table:**

Bruker APEXII DUO CCD area-detector diffractometer	2183 independent reflections
Radiation source: fine-focus sealed tube	1705 reflections with *I* > 2σ(*I*)
Graphite monochromator	*R*_int_ = 0.024
φ and ω scans	θ_max_ = 26.8°, θ_min_ = 1.7°
Absorption correction: multi-scan (*SADABS*; Bruker, 2012)	*h* = −8→8
*T*_min_ = 0.883, *T*_max_ = 0.985	*k* = −24→31
8322 measured reflections	*l* = −7→7

### (I) (2E,2'E)-3,3'-(1,4-Phenylene)bis[1-(2-methoxyphenyl)prop-2-en-1-one] Refinement

**Table:**

Refinement on *F*^2^	Primary atom site location: structure-invariant direct methods
Least-squares matrix: full	Hydrogen site location: inferred from neighbouring sites
*R*[*F*^2^ > 2σ(*F*^2^)] = 0.040	H-atom parameters constrained
*wR*(*F*^2^) = 0.119	*w* = 1/[σ^2^(*F*_o_^2^) + (0.0541*P*)^2^ + 0.2315*P*] where *P* = (*F*_o_^2^ + 2*F*_c_^2^)/3
*S* = 1.03	(Δ/σ)_max_ < 0.001
2183 reflections	Δρ_max_ = 0.19 e Å^−^^3^
137 parameters	Δρ_min_ = −0.14 e Å^−^^3^
0 restraints	

### (I) (2E,2'E)-3,3'-(1,4-Phenylene)bis[1-(2-methoxyphenyl)prop-2-en-1-one] Special details

**Table:**

Geometry. All esds (except the esd in the dihedral angle between two l.s. planes) are estimated using the full covariance matrix. The cell esds are taken into account individually in the estimation of esds in distances, angles and torsion angles; correlations between esds in cell parameters are only used when they are defined by crystal symmetry. An approximate (isotropic) treatment of cell esds is used for estimating esds involving l.s. planes.

### (I) (2E,2'E)-3,3'-(1,4-Phenylene)bis[1-(2-methoxyphenyl)prop-2-en-1-one] Fractional atomic coordinates and isotropic or equivalent isotropic displacement parameters (Å^2^)

**Table:**

	*x*	*y*	*z*	*U*_iso_*/*U*_eq_	
O1	0.51518 (16)	0.40818 (5)	0.74340 (18)	0.0615 (4)	
O2	0.57299 (19)	0.32593 (5)	0.2133 (2)	0.0652 (4)	
C1	0.7420 (2)	0.33535 (6)	0.3621 (3)	0.0492 (4)	
C2	0.9148 (3)	0.31024 (8)	0.3517 (4)	0.0689 (5)	
H2A	0.9237	0.2877	0.2307	0.083*	
C3	1.0731 (3)	0.31908 (9)	0.5223 (4)	0.0802 (6)	
H3A	1.1891	0.3026	0.5138	0.096*	
C4	1.0640 (3)	0.35160 (8)	0.7040 (4)	0.0731 (6)	
H4A	1.1715	0.3565	0.8194	0.088*	
C5	0.8930 (2)	0.37687 (7)	0.7125 (3)	0.0545 (4)	
H5A	0.8854	0.3988	0.8357	0.065*	
C6	0.7315 (2)	0.37030 (6)	0.5413 (2)	0.0420 (3)	
C7	0.5545 (2)	0.40138 (6)	0.5584 (2)	0.0426 (3)	
C8	0.4371 (2)	0.42573 (6)	0.3525 (2)	0.0430 (3)	
H8A	0.4840	0.4266	0.2198	0.052*	
C9	0.2652 (2)	0.44645 (6)	0.3551 (2)	0.0416 (3)	
H9A	0.2239	0.4435	0.4909	0.050*	
C10	0.13276 (19)	0.47340 (5)	0.1704 (2)	0.0379 (3)	
C11	−0.0569 (2)	0.48284 (6)	0.1921 (2)	0.0417 (3)	
H11A	−0.0958	0.4713	0.3222	0.050*	
C12	0.18710 (19)	0.49116 (6)	−0.0255 (2)	0.0413 (3)	
H12A	0.3122	0.4854	−0.0442	0.050*	
C13	0.5741 (4)	0.29130 (8)	0.0256 (3)	0.0783 (6)	
H13A	0.4472	0.2896	−0.0672	0.117*	
H13B	0.6140	0.2554	0.0785	0.117*	
H13C	0.6619	0.3054	−0.0614	0.117*	

### (I) (2E,2'E)-3,3'-(1,4-Phenylene)bis[1-(2-methoxyphenyl)prop-2-en-1-one] Atomic displacement parameters (Å^2^)

**Table:**

	*U*^11^	*U*^22^	*U*^33^	*U*^12^	*U*^13^	*U*^23^
O1	0.0571 (7)	0.0849 (9)	0.0427 (6)	0.0192 (6)	0.0106 (5)	0.0041 (6)
O2	0.0741 (8)	0.0619 (8)	0.0550 (7)	0.0175 (6)	0.0022 (6)	−0.0104 (6)
C1	0.0528 (9)	0.0429 (8)	0.0539 (9)	0.0073 (7)	0.0153 (7)	0.0099 (7)
C2	0.0705 (12)	0.0593 (11)	0.0838 (13)	0.0179 (9)	0.0322 (11)	0.0019 (9)
C3	0.0479 (11)	0.0703 (13)	0.1266 (19)	0.0173 (9)	0.0276 (12)	0.0082 (13)
C4	0.0414 (9)	0.0636 (12)	0.1088 (16)	0.0044 (8)	0.0027 (10)	0.0043 (11)
C5	0.0437 (8)	0.0469 (9)	0.0693 (11)	0.0002 (7)	0.0032 (7)	0.0037 (7)
C6	0.0406 (7)	0.0372 (7)	0.0488 (8)	0.0027 (6)	0.0107 (6)	0.0094 (6)
C7	0.0398 (7)	0.0430 (8)	0.0448 (8)	0.0017 (6)	0.0084 (6)	0.0031 (6)
C8	0.0436 (8)	0.0441 (8)	0.0421 (8)	0.0055 (6)	0.0108 (6)	0.0051 (6)
C9	0.0436 (8)	0.0444 (8)	0.0369 (7)	0.0047 (6)	0.0082 (6)	0.0003 (6)
C10	0.0388 (7)	0.0374 (7)	0.0371 (7)	0.0033 (5)	0.0071 (5)	−0.0034 (5)
C11	0.0422 (7)	0.0478 (8)	0.0370 (7)	0.0042 (6)	0.0127 (6)	0.0026 (6)
C12	0.0339 (7)	0.0485 (8)	0.0430 (7)	0.0043 (6)	0.0116 (6)	−0.0003 (6)
C13	0.1169 (18)	0.0615 (12)	0.0537 (11)	0.0150 (11)	0.0114 (11)	−0.0064 (9)

### (I) (2E,2'E)-3,3'-(1,4-Phenylene)bis[1-(2-methoxyphenyl)prop-2-en-1-one] Geometric parameters (Å, º)

**Table:**

O1—C7	1.2187 (17)	C7—C8	1.474 (2)
O2—C1	1.364 (2)	C8—C9	1.3267 (19)
O2—C13	1.419 (2)	C8—H8A	0.9300
C1—C2	1.387 (2)	C9—C10	1.4616 (19)
C1—C6	1.396 (2)	C9—H9A	0.9300
C2—C3	1.378 (3)	C10—C12	1.3897 (19)
C2—H2A	0.9300	C10—C11	1.4002 (19)
C3—C4	1.370 (3)	C11—C12^i^	1.3760 (19)
C3—H3A	0.9300	C11—H11A	0.9300
C4—C5	1.375 (2)	C12—C11^i^	1.3761 (19)
C4—H4A	0.9300	C12—H12A	0.9300
C5—C6	1.387 (2)	C13—H13A	0.9600
C5—H5A	0.9300	C13—H13B	0.9600
C6—C7	1.4932 (19)	C13—H13C	0.9600
			
C1—O2—C13	118.66 (14)	C9—C8—C7	120.50 (13)
O2—C1—C2	124.22 (16)	C9—C8—H8A	119.8
O2—C1—C6	115.79 (13)	C7—C8—H8A	119.8
C2—C1—C6	119.87 (16)	C8—C9—C10	127.89 (13)
C3—C2—C1	119.34 (18)	C8—C9—H9A	116.1
C3—C2—H2A	120.3	C10—C9—H9A	116.1
C1—C2—H2A	120.3	C12—C10—C11	118.03 (13)
C4—C3—C2	121.72 (17)	C12—C10—C9	122.99 (12)
C4—C3—H3A	119.1	C11—C10—C9	118.97 (12)
C2—C3—H3A	119.1	C12^i^—C11—C10	121.57 (12)
C3—C4—C5	118.73 (19)	C12^i^—C11—H11A	119.2
C3—C4—H4A	120.6	C10—C11—H11A	119.2
C5—C4—H4A	120.6	C11^i^—C12—C10	120.40 (12)
C4—C5—C6	121.43 (18)	C11^i^—C12—H12A	119.8
C4—C5—H5A	119.3	C10—C12—H12A	119.8
C6—C5—H5A	119.3	O2—C13—H13A	109.5
C5—C6—C1	118.81 (14)	O2—C13—H13B	109.5
C5—C6—C7	117.92 (14)	H13A—C13—H13B	109.5
C1—C6—C7	123.27 (14)	O2—C13—H13C	109.5
O1—C7—C8	121.49 (13)	H13A—C13—H13C	109.5
O1—C7—C6	119.30 (13)	H13B—C13—H13C	109.5
C8—C7—C6	119.12 (12)		
			
C13—O2—C1—C2	−4.7 (2)	C5—C6—C7—O1	−36.6 (2)
C13—O2—C1—C6	179.21 (14)	C1—C6—C7—O1	143.60 (15)
O2—C1—C2—C3	−174.24 (17)	C5—C6—C7—C8	140.06 (15)
C6—C1—C2—C3	1.7 (3)	C1—C6—C7—C8	−39.8 (2)
C1—C2—C3—C4	0.8 (3)	O1—C7—C8—C9	−13.5 (2)
C2—C3—C4—C5	−1.5 (3)	C6—C7—C8—C9	169.90 (14)
C3—C4—C5—C6	−0.4 (3)	C7—C8—C9—C10	178.03 (14)
C4—C5—C6—C1	2.8 (2)	C8—C9—C10—C12	−13.8 (2)
C4—C5—C6—C7	−177.02 (15)	C8—C9—C10—C11	167.44 (15)
O2—C1—C6—C5	172.83 (13)	C12—C10—C11—C12^i^	0.1 (2)
C2—C1—C6—C5	−3.4 (2)	C9—C10—C11—C12^i^	178.90 (13)
O2—C1—C6—C7	−7.4 (2)	C11—C10—C12—C11^i^	−0.1 (2)
C2—C1—C6—C7	176.38 (14)	C9—C10—C12—C11^i^	−178.84 (13)

Symmetry code: (i) −*x*, −*y*+1, −*z*.

### (II) (2E,2'E)-3,3'-(1,4-Phenylene)bis[1-(3-methoxyphenyl)prop-2-en-1-one] Crystal data

**Table:**

C_26_H_22_O_4_	*F*(000) = 420
*M**_r_* = 398.43	*D*_x_ = 1.305 Mg m^−^^3^
Monoclinic, *P*2_1_/*c*	Mo *K*α radiation, λ = 0.71073 Å
*a* = 5.2626 (5) Å	Cell parameters from 2940 reflections
*b* = 15.6157 (14) Å	θ = 2.6–27.7°
*c* = 12.4824 (11) Å	µ = 0.09 mm^−^^1^
β = 98.760 (2)°	*T* = 294 K
*V* = 1013.83 (16) Å^3^	Needle, yellow
*Z* = 2	0.90 × 0.48 × 0.09 mm

### (II) (2E,2'E)-3,3'-(1,4-Phenylene)bis[1-(3-methoxyphenyl)prop-2-en-1-one] Data collection

**Table:**

Bruker APEXII DUO CCD area-detector diffractometer	2458 independent reflections
Radiation source: fine-focus sealed tube	1574 reflections with *I* > 2σ(*I*)
Graphite monochromator	*R*_int_ = 0.043
φ and ω scans	θ_max_ = 28.1°, θ_min_ = 2.1°
Absorption correction: multi-scan (*SADABS*; Bruker, 2012)	*h* = −6→6
*T*_min_ = 0.874, *T*_max_ = 0.992	*k* = −20→20
17791 measured reflections	*l* = −16→16

### (II) (2E,2'E)-3,3'-(1,4-Phenylene)bis[1-(3-methoxyphenyl)prop-2-en-1-one] Refinement

**Table:**

Refinement on *F*^2^	Primary atom site location: structure-invariant direct methods
Least-squares matrix: full	Hydrogen site location: inferred from neighbouring sites
*R*[*F*^2^ > 2σ(*F*^2^)] = 0.044	H-atom parameters constrained
*wR*(*F*^2^) = 0.132	*w* = 1/[σ^2^(*F*_o_^2^) + (0.0567*P*)^2^ + 0.138*P*] where *P* = (*F*_o_^2^ + 2*F*_c_^2^)/3
*S* = 1.03	(Δ/σ)_max_ < 0.001
2458 reflections	Δρ_max_ = 0.14 e Å^−^^3^
137 parameters	Δρ_min_ = −0.14 e Å^−^^3^
0 restraints	

### (II) (2E,2'E)-3,3'-(1,4-Phenylene)bis[1-(3-methoxyphenyl)prop-2-en-1-one] Special details

**Table:**

Geometry. All esds (except the esd in the dihedral angle between two l.s. planes) are estimated using the full covariance matrix. The cell esds are taken into account individually in the estimation of esds in distances, angles and torsion angles; correlations between esds in cell parameters are only used when they are defined by crystal symmetry. An approximate (isotropic) treatment of cell esds is used for estimating esds involving l.s. planes.

### (II) (2E,2'E)-3,3'-(1,4-Phenylene)bis[1-(3-methoxyphenyl)prop-2-en-1-one] Fractional atomic coordinates and isotropic or equivalent isotropic displacement parameters (Å^2^)

**Table:**

	*x*	*y*	*z*	*U*_iso_*/*U*_eq_	
O1	1.3466 (2)	0.50160 (7)	0.83740 (9)	0.0753 (4)	
O2	2.0704 (2)	0.69588 (8)	1.00094 (10)	0.0771 (4)	
C1	1.6959 (3)	0.63162 (9)	0.89251 (12)	0.0502 (4)	
H1A	1.6962	0.5856	0.9399	0.060*	
C2	1.8795 (3)	0.69465 (10)	0.91308 (12)	0.0552 (4)	
C3	1.8786 (3)	0.76279 (11)	0.84322 (15)	0.0680 (5)	
H3A	2.0034	0.8052	0.8572	0.082*	
C4	1.6943 (3)	0.76859 (11)	0.75284 (16)	0.0723 (5)	
H4A	1.6945	0.8150	0.7062	0.087*	
C5	1.5086 (3)	0.70582 (10)	0.73089 (13)	0.0583 (4)	
H5A	1.3841	0.7100	0.6697	0.070*	
C6	1.5086 (3)	0.63679 (9)	0.80022 (11)	0.0461 (3)	
C7	1.3200 (3)	0.56519 (9)	0.78002 (11)	0.0499 (4)	
C8	1.1041 (3)	0.57169 (10)	0.69022 (12)	0.0525 (4)	
H8A	1.0879	0.6207	0.6473	0.063*	
C9	0.9345 (3)	0.51020 (9)	0.66922 (11)	0.0505 (4)	
H9A	0.9579	0.4629	0.7149	0.061*	
C10	0.7126 (2)	0.50652 (9)	0.58281 (10)	0.0450 (3)	
C11	0.6491 (3)	0.57310 (9)	0.50998 (11)	0.0514 (4)	
H11A	0.7478	0.6228	0.5161	0.062*	
C12	0.5587 (3)	0.43363 (10)	0.57116 (11)	0.0522 (4)	
H12A	0.5971	0.3884	0.6194	0.063*	
C13	2.1228 (4)	0.61900 (12)	1.05960 (15)	0.0766 (6)	
H13A	2.2657	0.6275	1.1163	0.115*	
H13B	2.1640	0.5748	1.0117	0.115*	
H13C	1.9743	0.6024	1.0908	0.115*	

### (II) (2E,2'E)-3,3'-(1,4-Phenylene)bis[1-(3-methoxyphenyl)prop-2-en-1-one] Atomic displacement parameters (Å^2^)

**Table:**

	*U*^11^	*U*^22^	*U*^33^	*U*^12^	*U*^13^	*U*^23^
O1	0.0691 (8)	0.0679 (7)	0.0765 (8)	−0.0179 (6)	−0.0289 (6)	0.0158 (6)
O2	0.0662 (8)	0.0728 (8)	0.0805 (8)	−0.0176 (6)	−0.0268 (6)	−0.0033 (6)
C1	0.0443 (8)	0.0523 (8)	0.0509 (8)	−0.0004 (6)	−0.0027 (6)	−0.0046 (6)
C2	0.0451 (8)	0.0557 (9)	0.0609 (9)	−0.0035 (7)	−0.0040 (7)	−0.0106 (7)
C3	0.0536 (10)	0.0573 (10)	0.0894 (12)	−0.0104 (8)	−0.0009 (9)	−0.0014 (9)
C4	0.0619 (11)	0.0631 (10)	0.0882 (13)	−0.0042 (8)	−0.0010 (9)	0.0156 (9)
C5	0.0494 (9)	0.0604 (9)	0.0612 (9)	0.0040 (7)	−0.0042 (7)	0.0039 (7)
C6	0.0369 (7)	0.0511 (8)	0.0480 (7)	0.0035 (6)	−0.0007 (6)	−0.0063 (6)
C7	0.0418 (8)	0.0558 (8)	0.0486 (8)	0.0010 (6)	−0.0044 (6)	−0.0040 (7)
C8	0.0435 (8)	0.0583 (8)	0.0513 (8)	0.0010 (7)	−0.0072 (6)	−0.0015 (6)
C9	0.0450 (8)	0.0561 (8)	0.0464 (7)	0.0026 (6)	−0.0063 (6)	−0.0029 (6)
C10	0.0366 (7)	0.0524 (8)	0.0432 (7)	0.0042 (6)	−0.0031 (6)	−0.0071 (6)
C11	0.0442 (8)	0.0511 (8)	0.0550 (8)	−0.0035 (6)	−0.0048 (7)	−0.0032 (6)
C12	0.0453 (8)	0.0549 (8)	0.0516 (8)	0.0015 (6)	−0.0076 (6)	0.0035 (6)
C13	0.0648 (11)	0.0864 (13)	0.0689 (11)	−0.0082 (9)	−0.0214 (9)	0.0006 (9)

### (II) (2E,2'E)-3,3'-(1,4-Phenylene)bis[1-(3-methoxyphenyl)prop-2-en-1-one] Geometric parameters (Å, º)

**Table:**

O1—C7	1.2199 (17)	C7—C8	1.4729 (18)
O2—C2	1.3698 (17)	C8—C9	1.310 (2)
O2—C13	1.411 (2)	C8—H8A	0.9300
C1—C2	1.376 (2)	C9—C10	1.4652 (17)
C1—C6	1.3998 (18)	C9—H9A	0.9300
C1—H1A	0.9300	C10—C11	1.3882 (19)
C2—C3	1.375 (2)	C10—C12	1.392 (2)
C3—C4	1.374 (2)	C11—C12^i^	1.3765 (18)
C3—H3A	0.9300	C11—H11A	0.9300
C4—C5	1.382 (2)	C12—C11^i^	1.3766 (18)
C4—H4A	0.9300	C12—H12A	0.9300
C5—C6	1.382 (2)	C13—H13A	0.9600
C5—H5A	0.9300	C13—H13B	0.9600
C6—C7	1.491 (2)	C13—H13C	0.9600
			
C2—O2—C13	117.70 (12)	C9—C8—C7	121.65 (14)
C2—C1—C6	119.92 (14)	C9—C8—H8A	119.2
C2—C1—H1A	120.0	C7—C8—H8A	119.2
C6—C1—H1A	120.0	C8—C9—C10	128.20 (14)
O2—C2—C3	115.34 (13)	C8—C9—H9A	115.9
O2—C2—C1	124.61 (14)	C10—C9—H9A	115.9
C3—C2—C1	120.05 (14)	C11—C10—C12	117.76 (12)
C4—C3—C2	120.36 (15)	C11—C10—C9	122.57 (13)
C4—C3—H3A	119.8	C12—C10—C9	119.67 (13)
C2—C3—H3A	119.8	C12^i^—C11—C10	120.65 (13)
C3—C4—C5	120.36 (16)	C12^i^—C11—H11A	119.7
C3—C4—H4A	119.8	C10—C11—H11A	119.7
C5—C4—H4A	119.8	C11^i^—C12—C10	121.59 (13)
C4—C5—C6	119.78 (14)	C11^i^—C12—H12A	119.2
C4—C5—H5A	120.1	C10—C12—H12A	119.2
C6—C5—H5A	120.1	O2—C13—H13A	109.5
C5—C6—C1	119.53 (13)	O2—C13—H13B	109.5
C5—C6—C7	122.86 (12)	H13A—C13—H13B	109.5
C1—C6—C7	117.59 (13)	O2—C13—H13C	109.5
O1—C7—C8	120.62 (13)	H13A—C13—H13C	109.5
O1—C7—C6	119.81 (12)	H13B—C13—H13C	109.5
C8—C7—C6	119.57 (13)		
			
C13—O2—C2—C3	−164.12 (17)	C1—C6—C7—O1	−6.5 (2)
C13—O2—C2—C1	16.2 (2)	C5—C6—C7—C8	−7.6 (2)
C6—C1—C2—O2	179.81 (14)	C1—C6—C7—C8	173.97 (13)
C6—C1—C2—C3	0.2 (2)	O1—C7—C8—C9	0.2 (2)
O2—C2—C3—C4	−179.41 (16)	C6—C7—C8—C9	179.71 (14)
C1—C2—C3—C4	0.3 (3)	C7—C8—C9—C10	−179.20 (14)
C2—C3—C4—C5	−0.3 (3)	C8—C9—C10—C11	−1.7 (2)
C3—C4—C5—C6	0.0 (3)	C8—C9—C10—C12	177.64 (16)
C4—C5—C6—C1	0.5 (2)	C12—C10—C11—C12^i^	−0.4 (2)
C4—C5—C6—C7	−177.93 (16)	C9—C10—C11—C12^i^	178.90 (14)
C2—C1—C6—C5	−0.5 (2)	C11—C10—C12—C11^i^	0.4 (2)
C2—C1—C6—C7	177.94 (14)	C9—C10—C12—C11^i^	−178.91 (14)
C5—C6—C7—O1	171.91 (15)		

Symmetry code: (i) −*x*+1, −*y*+1, −*z*+1.

### (III) (2E,2'E)-3,3'-(1,4-Phenylene)bis[1-(3,4-dimethoxyphenyl)prop-2-en-1-one] Crystal data

**Table:**

C_28_H_26_O_6_	*F*(000) = 484
*M**_r_* = 458.49	*D*_x_ = 1.285 Mg m^−^^3^
Monoclinic, *P*2_1_/*n*	Mo *K*α radiation, λ = 0.71073 Å
*a* = 6.9595 (6) Å	Cell parameters from 3549 reflections
*b* = 21.0272 (17) Å	θ = 2.7–28.5°
*c* = 8.3297 (7) Å	µ = 0.09 mm^−^^1^
β = 103.602 (2)°	*T* = 294 K
*V* = 1184.77 (17) Å^3^	Block, yellow
*Z* = 2	0.35 × 0.27 × 0.16 mm

### (III) (2E,2'E)-3,3'-(1,4-Phenylene)bis[1-(3,4-dimethoxyphenyl)prop-2-en-1-one] Data collection

**Table:**

Bruker APEXII DUO CCD area-detector diffractometer	3165 independent reflections
Radiation source: fine-focus sealed tube	2328 reflections with *I* > 2σ(*I*)
Graphite monochromator	*R*_int_ = 0.030
φ and ω scans	θ_max_ = 29.0°, θ_min_ = 1.9°
Absorption correction: multi-scan (*SADABS*; Bruker, 2012)	*h* = −9→8
*T*_min_ = 0.890, *T*_max_ = 0.985	*k* = −28→26
12532 measured reflections	*l* = −11→11

### (III) (2E,2'E)-3,3'-(1,4-Phenylene)bis[1-(3,4-dimethoxyphenyl)prop-2-en-1-one] Refinement

**Table:**

Refinement on *F*^2^	Primary atom site location: structure-invariant direct methods
Least-squares matrix: full	Hydrogen site location: inferred from neighbouring sites
*R*[*F*^2^ > 2σ(*F*^2^)] = 0.049	H-atom parameters constrained
*wR*(*F*^2^) = 0.136	*w* = 1/[σ^2^(*F*_o_^2^) + (0.0557*P*)^2^ + 0.2808*P*] where *P* = (*F*_o_^2^ + 2*F*_c_^2^)/3
*S* = 1.04	(Δ/σ)_max_ < 0.001
3165 reflections	Δρ_max_ = 0.20 e Å^−^^3^
156 parameters	Δρ_min_ = −0.16 e Å^−^^3^
0 restraints	

### (III) (2E,2'E)-3,3'-(1,4-Phenylene)bis[1-(3,4-dimethoxyphenyl)prop-2-en-1-one] Special details

**Table:**

Geometry. All esds (except the esd in the dihedral angle between two l.s. planes) are estimated using the full covariance matrix. The cell esds are taken into account individually in the estimation of esds in distances, angles and torsion angles; correlations between esds in cell parameters are only used when they are defined by crystal symmetry. An approximate (isotropic) treatment of cell esds is used for estimating esds involving l.s. planes.

### (III) (2E,2'E)-3,3'-(1,4-Phenylene)bis[1-(3,4-dimethoxyphenyl)prop-2-en-1-one] Fractional atomic coordinates and isotropic or equivalent isotropic displacement parameters (Å^2^)

**Table:**

	*x*	*y*	*z*	*U*_iso_*/*U*_eq_	
O1	0.26810 (16)	0.04480 (7)	0.05640 (13)	0.0667 (4)	
O2	−0.37696 (15)	0.15896 (5)	0.01870 (14)	0.0560 (3)	
O3	−0.33472 (18)	0.22511 (6)	0.28240 (17)	0.0694 (4)	
C1	−0.04399 (19)	0.11677 (6)	0.10365 (16)	0.0377 (3)	
H1A	−0.0610	0.0919	0.0090	0.045*	
C2	−0.1959 (2)	0.15447 (6)	0.12601 (17)	0.0399 (3)	
C3	−0.1726 (2)	0.19126 (7)	0.2696 (2)	0.0459 (3)	
C4	0.0049 (2)	0.19044 (7)	0.3851 (2)	0.0525 (4)	
H4A	0.0217	0.2154	0.4796	0.063*	
C5	0.1585 (2)	0.15265 (7)	0.36105 (18)	0.0469 (4)	
H5A	0.2779	0.1525	0.4398	0.056*	
C6	0.13686 (19)	0.11510 (6)	0.22134 (16)	0.0369 (3)	
C7	0.2924 (2)	0.07224 (7)	0.18910 (16)	0.0413 (3)	
C8	0.4765 (2)	0.06233 (7)	0.31697 (16)	0.0419 (3)	
H8A	0.4879	0.0794	0.4218	0.050*	
C9	0.62488 (19)	0.02971 (7)	0.28517 (16)	0.0385 (3)	
H9A	0.6062	0.0140	0.1782	0.046*	
C10	0.81526 (18)	0.01514 (6)	0.39711 (15)	0.0348 (3)	
C11	0.8724 (2)	0.03954 (7)	0.55600 (16)	0.0422 (3)	
H11A	0.7872	0.0664	0.5947	0.051*	
C12	0.9466 (2)	−0.02452 (7)	0.34276 (16)	0.0429 (3)	
H12A	0.9117	−0.0412	0.2364	0.051*	
C13	−0.4046 (3)	0.12540 (11)	−0.1316 (2)	0.0727 (6)	
H13A	−0.5375	0.1316	−0.1950	0.109*	
H13B	−0.3816	0.0809	−0.1092	0.109*	
H13C	−0.3135	0.1409	−0.1929	0.109*	
C14	−0.3309 (3)	0.25570 (12)	0.4350 (3)	0.0920 (8)	
H14A	−0.4595	0.2727	0.4331	0.138*	
H14B	−0.2360	0.2896	0.4516	0.138*	
H14C	−0.2948	0.2255	0.5234	0.138*	

### (III) (2E,2'E)-3,3'-(1,4-Phenylene)bis[1-(3,4-dimethoxyphenyl)prop-2-en-1-one] Atomic displacement parameters (Å^2^)

**Table:**

	*U*^11^	*U*^22^	*U*^33^	*U*^12^	*U*^13^	*U*^23^
O1	0.0427 (6)	0.1049 (10)	0.0443 (6)	0.0272 (6)	−0.0066 (5)	−0.0257 (6)
O2	0.0389 (6)	0.0653 (7)	0.0565 (6)	0.0185 (5)	−0.0031 (5)	−0.0026 (5)
O3	0.0576 (7)	0.0680 (8)	0.0820 (9)	0.0234 (6)	0.0149 (6)	−0.0190 (7)
C1	0.0349 (7)	0.0413 (7)	0.0355 (6)	0.0040 (5)	0.0056 (5)	0.0013 (5)
C2	0.0344 (7)	0.0387 (7)	0.0443 (7)	0.0040 (5)	0.0048 (5)	0.0051 (6)
C3	0.0434 (8)	0.0380 (7)	0.0578 (9)	0.0059 (6)	0.0149 (7)	−0.0026 (6)
C4	0.0523 (9)	0.0492 (9)	0.0544 (9)	−0.0001 (7)	0.0092 (7)	−0.0169 (7)
C5	0.0378 (7)	0.0522 (8)	0.0469 (8)	−0.0014 (6)	0.0024 (6)	−0.0087 (7)
C6	0.0308 (6)	0.0414 (7)	0.0372 (6)	0.0006 (5)	0.0052 (5)	0.0015 (5)
C7	0.0306 (7)	0.0553 (8)	0.0357 (6)	0.0048 (6)	0.0030 (5)	−0.0022 (6)
C8	0.0327 (7)	0.0572 (8)	0.0325 (6)	0.0042 (6)	0.0010 (5)	−0.0027 (6)
C9	0.0303 (7)	0.0504 (8)	0.0316 (6)	0.0003 (5)	0.0010 (5)	0.0006 (5)
C10	0.0274 (6)	0.0443 (7)	0.0306 (6)	−0.0005 (5)	0.0026 (5)	0.0037 (5)
C11	0.0323 (7)	0.0559 (8)	0.0365 (7)	0.0091 (6)	0.0040 (5)	−0.0049 (6)
C12	0.0358 (7)	0.0574 (9)	0.0316 (6)	0.0054 (6)	0.0004 (5)	−0.0066 (6)
C13	0.0523 (11)	0.0953 (14)	0.0582 (10)	0.0179 (10)	−0.0117 (8)	−0.0150 (10)
C14	0.0722 (14)	0.0934 (16)	0.1191 (19)	0.0054 (12)	0.0398 (13)	−0.0499 (15)

### (III) (2E,2'E)-3,3'-(1,4-Phenylene)bis[1-(3,4-dimethoxyphenyl)prop-2-en-1-one] Geometric parameters (Å, º)

**Table:**

O1—C7	1.2229 (17)	C8—C9	1.318 (2)
O2—C2	1.3660 (16)	C8—H8A	0.9300
O2—C13	1.410 (2)	C9—C10	1.4621 (16)
O3—C3	1.3593 (18)	C9—H9A	0.9300
O3—C14	1.419 (2)	C10—C11	1.3877 (18)
C1—C2	1.3688 (19)	C10—C12	1.3893 (19)
C1—C6	1.4022 (18)	C11—C12^i^	1.3781 (18)
C1—H1A	0.9300	C11—H11A	0.9300
C2—C3	1.401 (2)	C12—C11^i^	1.3780 (18)
C3—C4	1.376 (2)	C12—H12A	0.9300
C4—C5	1.384 (2)	C13—H13A	0.9600
C4—H4A	0.9300	C13—H13B	0.9600
C5—C6	1.3848 (19)	C13—H13C	0.9600
C5—H5A	0.9300	C14—H14A	0.9600
C6—C7	1.4807 (19)	C14—H14B	0.9600
C7—C8	1.4756 (17)	C14—H14C	0.9600
			
C2—O2—C13	117.30 (12)	C7—C8—H8A	119.4
C3—O3—C14	117.82 (15)	C8—C9—C10	128.07 (12)
C2—C1—C6	120.95 (13)	C8—C9—H9A	116.0
C2—C1—H1A	119.5	C10—C9—H9A	116.0
C6—C1—H1A	119.5	C11—C10—C12	118.04 (11)
O2—C2—C1	125.04 (13)	C11—C10—C9	122.96 (12)
O2—C2—C3	115.10 (12)	C12—C10—C9	119.00 (12)
C1—C2—C3	119.85 (12)	C12^i^—C11—C10	120.96 (13)
O3—C3—C4	125.24 (14)	C12^i^—C11—H11A	119.5
O3—C3—C2	115.11 (13)	C10—C11—H11A	119.5
C4—C3—C2	119.65 (13)	C11^i^—C12—C10	121.00 (12)
C3—C4—C5	120.21 (14)	C11^i^—C12—H12A	119.5
C3—C4—H4A	119.9	C10—C12—H12A	119.5
C5—C4—H4A	119.9	O2—C13—H13A	109.5
C4—C5—C6	120.91 (13)	O2—C13—H13B	109.5
C4—C5—H5A	119.5	H13A—C13—H13B	109.5
C6—C5—H5A	119.5	O2—C13—H13C	109.5
C5—C6—C1	118.41 (12)	H13A—C13—H13C	109.5
C5—C6—C7	124.03 (12)	H13B—C13—H13C	109.5
C1—C6—C7	117.55 (12)	O3—C14—H14A	109.5
O1—C7—C8	119.85 (12)	O3—C14—H14B	109.5
O1—C7—C6	119.98 (12)	H14A—C14—H14B	109.5
C8—C7—C6	120.17 (12)	O3—C14—H14C	109.5
C9—C8—C7	121.13 (12)	H14A—C14—H14C	109.5
C9—C8—H8A	119.4	H14B—C14—H14C	109.5
			
C13—O2—C2—C1	−4.5 (2)	C2—C1—C6—C5	−0.1 (2)
C13—O2—C2—C3	176.83 (15)	C2—C1—C6—C7	178.99 (13)
C6—C1—C2—O2	−179.73 (13)	C5—C6—C7—O1	−173.70 (15)
C6—C1—C2—C3	−1.1 (2)	C1—C6—C7—O1	7.3 (2)
C14—O3—C3—C4	−8.5 (3)	C5—C6—C7—C8	6.5 (2)
C14—O3—C3—C2	170.79 (17)	C1—C6—C7—C8	−172.50 (13)
O2—C2—C3—O3	1.14 (19)	O1—C7—C8—C9	7.3 (2)
C1—C2—C3—O3	−177.63 (13)	C6—C7—C8—C9	−172.95 (14)
O2—C2—C3—C4	−179.52 (14)	C7—C8—C9—C10	−179.46 (13)
C1—C2—C3—C4	1.7 (2)	C8—C9—C10—C11	−5.6 (2)
O3—C3—C4—C5	178.13 (15)	C8—C9—C10—C12	174.89 (15)
C2—C3—C4—C5	−1.1 (2)	C12—C10—C11—C12^i^	−0.4 (2)
C3—C4—C5—C6	0.0 (2)	C9—C10—C11—C12^i^	−179.97 (14)
C4—C5—C6—C1	0.7 (2)	C11—C10—C12—C11^i^	0.4 (2)
C4—C5—C6—C7	−178.35 (14)	C9—C10—C12—C11^i^	179.98 (13)

Symmetry code: (i) −*x*+2, −*y*, −*z*+1.
